# A randomized controlled trial study protocol for Xiao-Qing-Long decoction in the treatment of refractory asthma: Study protocol clinical trial (spirit compliant)

**DOI:** 10.1097/MD.0000000000018911

**Published:** 2020-01-31

**Authors:** Hongjing Yang, Chuantao Zhang, Wenfan Gan, Jun Chen, Jianying Wu, Wei Xiao, Yang Yang, Keni Zhao, Zengtao Sun, Xiaohong Xie, Qingsong Huang

**Affiliations:** a Department of Respiratory Medicine; b Department of Critical Care Medicine; c Department of Gastroenterology, Hospital of Chengdu University of Traditional Chinese Medicine, Chengdu, China.

**Keywords:** refractory asthma, Chinese herbal medicine, randomized controlled trial, intestinal microbiota, Xiao-Qing-Long decoction

## Abstract

**Introduction:**

People with refractory asthma (RA) often seek help from Chinese medicine due to dissatisfaction with conventional treatments. External cold and internal fluid syndrome is the most common type of asthma and the Chinese herbal medicine formula Xiao-Qing-Long (XQL) decoction is commonly prescribed for patients with asthma with this syndrome. However, there is no direct evidence to support the efficacy and safety of XQL decoction for RA treatment and its potential mechanism is still unclear.

**Methods:**

We propose a double-blind, placebo-controlled, randomized superiority trial. After a 2-week run-in period, 112 eligible participants will be recruited and randomly allocated to an experimental group or control group in a 1:1 ratio. Patients in the experimental group will take XQL decoction, while patients in the control group will receive a matched placebo. Symbicort Turbuhaler and Montelukast sodium tablets will be provided as the basic treatment for the 2 groups. All participants will receive 4 weeks of treatment and 12 weeks of follow-up. The primary outcome is the mean change in the asthma control test score from the baseline to 4 weeks posttreatment. The secondary outcomes include quality of life, lung function, curative effect of traditional Chinese medicine, and rescue medication used. This trial will also include analyses of the associations between intestinal microbiota and RA treatment. Any side effects of the treatment will be recorded.

**Discussion:**

The results of this trial will provide consolidated evidence of the effect of XQL decoction for RA and the potential mechanism by which XQL decoction acts, which will inform treatment options for patients with RA.

## Abstract 2

Supplemental Digital Content is available in the text

## 1 Introduction

Refractory asthma (RA) is asthma that is uncontrolled despite Global Initiative for Asthma (GINA) Step 4 or 5 treatment, or that requires such treatment to maintain good symptom control and reduce the risk of exacerbation.^[1]^ There are currently 300 million patients with asthma in the world,^[1]^ of which 45.7 million are in China.^[2]^ RA accounts for 3% to 10% of all patients with asthma,^[3,4]^ but the emergency visit and hospitalization rates are 15 times and 20 times higher than those for patients with mild and moderate asthma,^[5]^ respectively. Medical costs for patients with RA account for more than 60% of asthma-related costs.^[6]^ In addition, the symptoms, aggravation, and drug side effects associated with RA can have a significant impact on patients’ mental and emotional health, interpersonal relationships, and careers, resulting in a considerable disease burden.^[7]^

The treatment of RA still relies heavily on the maximal optimal use of corticosteroids and bronchodilators.^[1]^ Although long-term oral systemic corticosteroids may be effective, they have severe adverse effects that limit their use.^[8]^ Other therapies recommended for RA include leukotriene regulators, immunosuppressants, antimetabolic drugs,^[1]^ biologic targeted therapies,^[9,10]^ and bronchial thermoplasty.^[11]^ Due to a lack of high-level evidence-based medical research, the efficacy and safety of these drugs and therapies are not clear. There is an urgent need for new treatment plans to improve the overall control and prognosis of RA and reduce medical costs.

Due to the recurrence of asthma and the lack of satisfactory treatments, many sufferers seek Chinese herbal medicine (CHM) as a complementary and alternative medicine.^[12]^ The use of CHM in patients with asthma ranges from 4% to 79%.^[13]^ CHM is reportedly effective in treating chronic persistent asthma,^[14]^ despite the lack of recommendations in international guidelines. A recently published systematic review^[15]^ included 29 randomized controlled trials and showed that herbs can improve lung function and asthma control. Among the CHM formula used for asthma, XQL decoction is the most frequently prescribed. XQL decoction is a classic prescription first recorded in the “Treatise on Febrile Diseases” by Zhang Zhongjing, a famous physician of the Eastern Han Dynasty, and has been used for nearly 2000 years. The formula comprises 8 herbs including Ephedrae Herba (Ma huang), Cinnamomi Ramulus (Gui zhi), Paeoniae Radix Rubra (Chi shao), Schisandrae Chinensis Fructus (Wu wei zi), Zingiberis Rhizoma (Gan jiang), Asari Radix Et Rhizoma (Xi xin), Rhizoma Pinelliae Praeparata (Fa ban xia), and Glycyrrhizae Radix Et Rhizoma (Zhi gan cao). XQL decoction can relieve exterior syndrome (*Jie Biao*), as well as dispel cold (*San Han*), eliminate phlegm (*Qu Tan*), and relieve asthma (*Ping Chuan*). Modern pharmacologic studies have shown that XQL decoction has multiple effects such as antiallergic,^[16]^ anti-inflammatory,^[17]^ antitussive,^[18]^ immune regulation,^[19]^ inhibition of airway remodeling, and expansion of bronchial smooth muscle.^[20,21]^ XQL decoction is widely used in the Chinese population for the treatment of asthma. Thus, it is reasonable to hypothesize that XQL decoction is effective for the treatment of RA. To date, however, there is no direct evidence to support its efficacy and safety.

Several studies have shown that a low diversity of intestinal microflora is closely related to a high risk of allergic disease.^[22,23]^ Recent studies have also confirmed that intestinal microflora play a role in the occurrence and development of asthma.^[24,25]^ Traditional Chinese medicine may alleviate the symptoms and related indicators of asthma to some extent by affecting the structure of intestinal flora.^[26]^ Based on these previous studies, we hypothesize that XQL decoction modulates intestine microorganisms and changes both intestinal microbiome composition and function, thereby reducing the symptoms of RA. To the best of our knowledge, changes in microbiome composition in the intestine of patients with RA treated with XQL decoction have not yet been fully characterized.

## 2 Methods and design

### 2.1 Design

**Figure 1:**
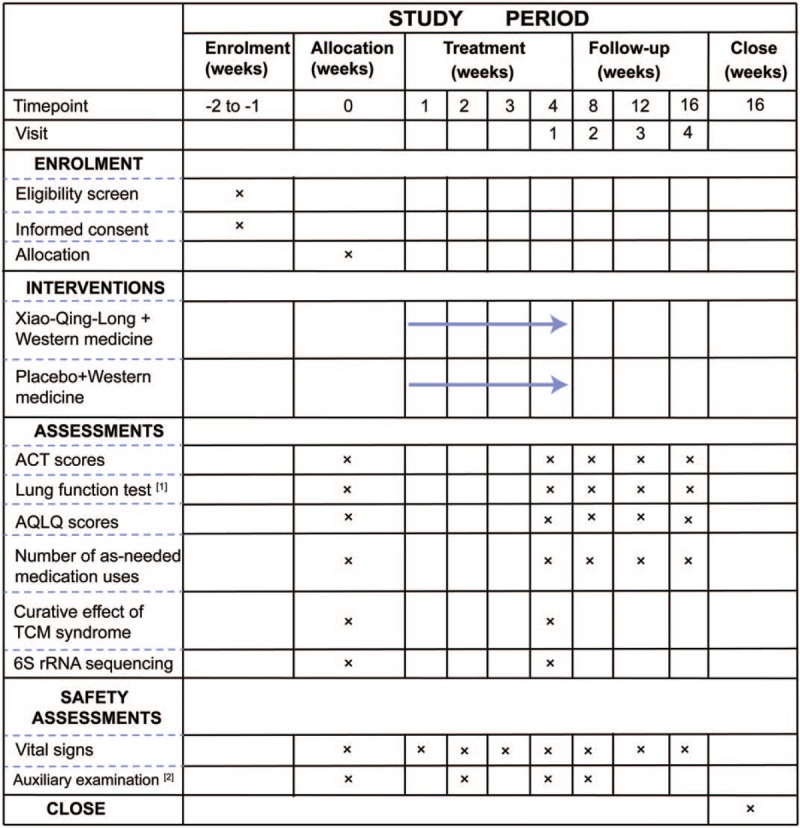
Spirit figure of enrollment, interventions, and assessments. [1] Lung function tests: FEV_1_ = forced expiratory volume in one second, FVC = forced volume capacity, PEF = peak expiratory flow. [2] Laboratory tests: blood, urine, feces, electrocardiogram, and kidney and liver function tests. ACT = asthma control test, AQLQ = Asthma Quality of Life Questionnaire, TCM = traditional Chinese medicine.

**Figure 2:**
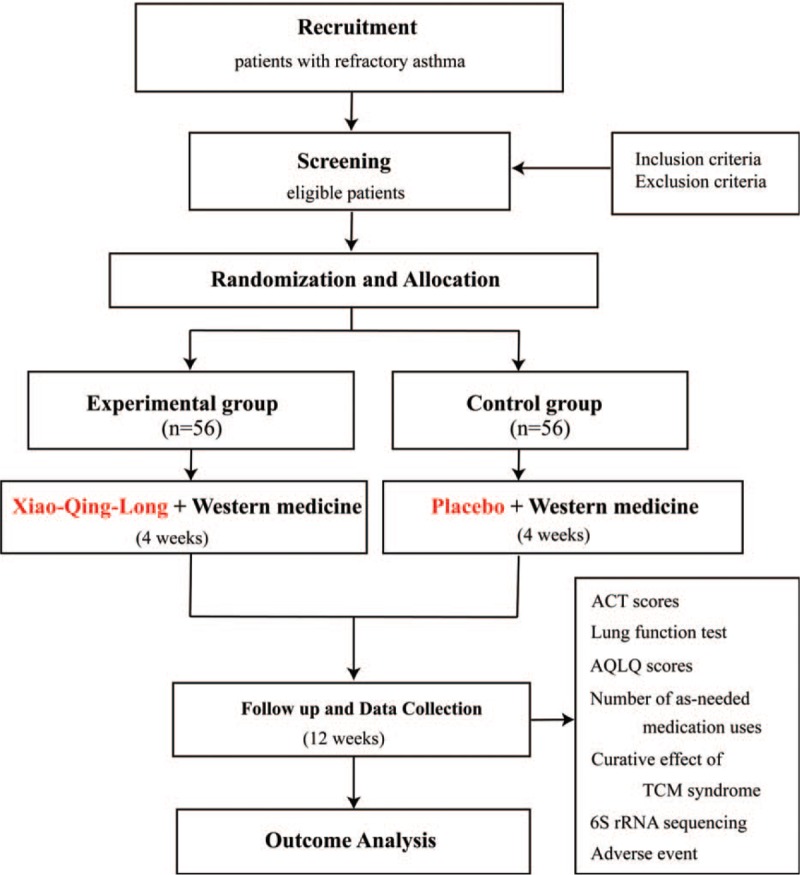
Flow chart of the study design. ACT = asthma control test, AQLQ = Asthma Quality of Life Questionnaire, TCM = traditional Chinese medicine.

This study is a prospective, double-blind, randomized, placebo-controlled clinical trial. This trial has been registered with the Chinese Clinical Trial Registry (no ChiCTR1900027760, registered November 27, 2019). After obtaining written informed consent, a 2-week run-in period will be implemented, after which 112 eligible participants will be randomly assigned to a XQL group or a control group in a 1:1 ratio. The 2 groups will then undergo a 4-week treatment and a 12-week follow-up period. The trial aims to investigate the additional benefits and safety of XQL decoction compared with western medicine alone for the treatment of RA, and to explore the mechanism in light of intestinal microecology. This study will adhere to the Standard Protocol Items: Recommendations for Interventional Trials (SPIRIT) 2013 statement (see Fig. 1 for the SPIRIT figure of enrollment, interventions, and assessments; and Additional File 1 for the SPIRIT checklist).^[27]^ The study flow chart is presented in Figure 2.

### 2.2 Ethics approval

The study is in compliance with the Declaration of Helsinki (Edinburgh 2000 version). The final amendments (version: November 4, 2019) and the consent form have been reviewed and approved by the China Ethics Committee of Registering Clinical Trials (approval no: ChiECRCT20190231). If there is any amendment to the protocol, approval must be again sought from the Ethics Committee.

### 2.3 Recruitment

Participants will be recruited via a local advertisement and doctor referrals from respiratory clinics at the hospital at Chengdu University of Traditional Chinese Medicine (Chengdu, China). Before enrollment, participants will be provided with detailed information about the clinical study, including its purpose, processing, scheduling, and possible risks and benefits. All patients will be required to sign an informed written consent before any study procedures commence.

### 2.4 Sample size

Sample size calculations are based on the primary outcome (asthma control test [ACT] score at 4 weeks). According to the previous literature,^[28]^ the expected average ACT score of the control group will be 8.67. At the 5% significance level, a total of 50 patients per group is required to achieve 80% power and to determine an increase of 2 points in the ACT score between the experimental group and control group, assuming that the standard deviation of the groups is equal to 4.^[29]^ The software Power Analysis and Sample Size version 11.0 (PASS 11.0) was used to calculate the sample sizes of the experimental group and the control group as 50 cases each. Thus, assuming that 10% of patients are likely to be lost during follow-up, a total of 112 patients will be enrolled.

### 2.5 Randomization and allocation concealment

A member of the Sichuan traditional Chinese medicine (TCM) evidence-based Medicine Center will generate 112 random serial numbers using SAS 9.2 software (SAS, Cary, NC). Randomization will occur after screening and baseline assessment and eligible patients with RA will be assigned to the experimental group or the control group in a 1:1 ratio. The group numbers will be provided in continuously numbered, sealed envelopes made from carbonless paper. The envelopes will be kept by a study administrator who will not directly participate in the recruitment or follow-up of any participant, and the group numbers will be subsequently disclosed. The administrator will open one envelope and provide the participant with their group number on the day of inclusion. Therefore, the participants, clinical practitioners, the outcome evaluators, the data manager, and statistician will not know the treatment allocations, which will not be revealed until the end of study.

### 2.6 Blinding

This trial is a double-blind design in which neither the researchers nor the participants will be aware of their treatment group during the trial period. Both herbal granules and placebo granules will be produced, packaged, and marked by Sichuan Green Pharmaceutical Technology Development Co, Ltd to ensure that they are consistent in appearance, shape, smell, and specifications. In addition, the research team will be instructed not to communicate with the participants regarding their possible treatment group allocation. Only in emergencies, such as serious adverse events, or if the patient needs emergency treatment, can the researchers report to the principal researcher to decide whether to expose the blind.

### 2.7 Diagnostic criteria

Participants must meet the western medicine diagnostic criteria for RA (Table 1)^[4]^ and the TCM syndrome diagnostic criteria of external cold and internal fluid syndrome (Table 2).^[30]^ The determination of syndrome differentiation shall be determined independently by 2 designated deputy physicians of TCM.

**Table 1:**
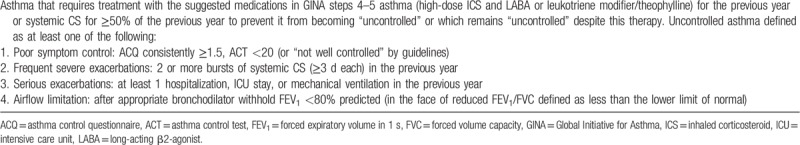
Western medicine diagnostic criteria for refractory asthma^[4]^.

**Table 2:**
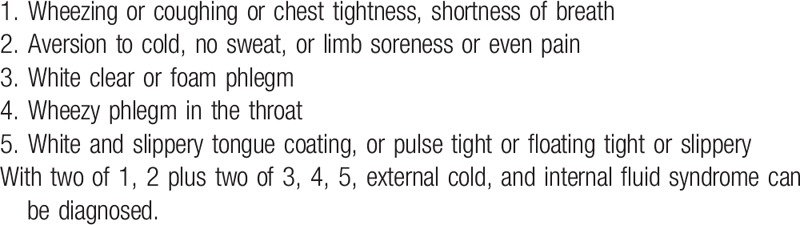
Diagnostic criteria for traditional Chinese medicine differentiation of external cold and internal fluid syndrome^[30]^.

### 2.8 Eligibility criteria

Inclusion criteria:

- Participants aged 18 to 65 years with a diagnosis of asthma for at least 1 year according to GINA criteria; no gender limitations.
- In accordance with the western medicine diagnostic criteria for RA, patients cannot achieve control of the disease even though they are following GINA Step 4 to 5 level recommended treatments (such as high dose inhaled corticosteroids [ICS], budesonide > 800 μg, fluticasone > 500 μg).
- Participants meet the criteria for external cold and internal fluid syndrome in TCM.
- Participants can complete this study and all tests.
- Participants provide informed written consent and volunteer to participate in the trial.

Exclusion criteria:

- Patients with coexistent pulmonary diseases (e.g., bronchiectasis, chronic obstructive pulmonary disease, tuberculosis, or interstitial lung disease).
- Patients with a clinically significant upper or lower respiratory infection within 4 weeks prior to study entry.
- Patients with severe comorbidities, or laboratory data suggesting severe systemic disease (such as liver and kidney insufficiency) or mental disorder.
- Women who are breastfeeding, pregnant, or preparing for pregnancy.
- Allergies for known drugs or experimental drugs in certain herbs.
- Patients have participated in other clinical trials in the 4 weeks preceding the trial.
- Patients have consumed yogurt or prebiotics for one month preceding the trial.
- Current or past smokers with a smoking history of at least 10 pack-years.
- Unwilling or unable to switch from current asthma treatment regimen.

### 2.9 Termination and withdrawal criteria

All participants will be told that they have the right to withdraw from the trial and that they will be provided with standardized treatment if they withdraw. The reason for the withdrawal will be recorded in their case report file (CRF). The criteria for stopping treatment and withdrawing patients from the research project are:

- the participant has suffered an AE related to taking the drug, and the investigator believes it is not appropriate for them to continue taking the drug;
- the participant develops another severe disease that needs to be treated during the study;
- the participant has suffered a severe exacerbation;
- poor compliance, such that the actual drug usage is <80% of the prescribed dose; and
- use of drugs prescribed for RA during the study.

### 2.10 Test drugs

Test drugs are XQL decoction and XQL mimetic agent (placebo), provided by the Sichuan Green Pharmaceutical Technology Development Co, Ltd (Sichuan, China). Whole ingredients of XQL decoction are Ephedrae Herba (Ma huang) 10 g, Cinnamomi Ramulus (Gui zhi) 10 g, Paeoniae Radix Rubra (Chi shao) 10 g, Schisandrae Chinensis Fructus (Wu wei zi) 15 g, Zingiberis Rhizoma (Gan jiang) 10 g, Asari Radix Et Rhizoma (Xi xin) 6 g, Rhizoma Pinelliae Praeparata (Fa ban xia) 15 g, and Glycyrrhizae Radix Et Rhizoma (Zhi gan cao) 10 g. The herbs in the prescription are mixed, cooked, filtered, and pressure spray-dried to form granules by pharmaceutical manufacturer. The granules are packaged into small single-dose sachets, each weighing 10 g. placebos consists of starch without active ingredients. By adding a variety of food colorings, the placebo is as close as possible in appearance and taste to the real granules.

## 3 Interventions

### 3.1 Treatment plan

Both groups will be treated with western medicine in accordance with GINA guidelines^[1]^: Budesonide and formoterol fumarate powder for inhalation (Symbicort Turbuhaler, 320 μg: 4.5 μg × 60 inhaled; AstraZeneca, Södartälje, Sweden), 324.5 μg every time, twice a day; Montelukast sodium tablets (Singulair, 10 mg × 30 tablets; Merck Sharp & Dohme Ltd, Hangzhou, China), 10 mg taken daily before bed. A salbutamol inhaler (Ventolin, 100 μg/puff; GlaxoSmithKline, London, UK) can be used as needed in the event of severe wheezing during the study period, with no more than 8 puffs a day. The participants will be required to record any first-aid medication in their patient diary. If symptoms are not relieved, the subjects will be required to contact physicians immediately to initiate further management according to GINA. At the same time, all participants will be taught how to use inhalers correctly, maintain good compliance, and prevent colds.

Experimental group: Participants in the experimental group will receive 10 g XQL granules twice daily for 4 weeks, after breakfast and supper, dissolving each dose in 100 mL warm boiled water.

Control group: Patients in the control group will be given 10 g placebo granules twice daily for 4 weeks. The measurements will be in accordance with the experimental group.

Concomitant asthma medications: Throughout the study, all patients can accept salbutamol as a rescue medication. However, patients are not allowed to receive other asthma-related medications or CHM.

### 3.2 Collection of stool

The subject intestinal samples will be collected by using the fecal collection bowl (to avoid urine mixing). Subsequently, the samples will be dipped with sterile cotton swabs and transferred to an aseptic freezing tube, frozen in liquid nitrogen for more than 4 hours, and then transferred to −80°C for preservation. All samples will be destructed after use.

### 3.3 Outcome measures

Primary outcome: The primary outcome is the change in ACT score from the baseline to the end of the treatment phase (week 4). ACT is a patient-based tool for identifying poorly controlled asthma and comprises 5 questions. The score is a reliable and effective measure of changes in asthma control over time in different patient populations.^[31]^

Secondary outcomes:

1. Health-related quality of life: The total score from the Asthma Quality of Life Questionnaire (AQLQ) will be used to assess the quality of life of the participants (at weeks 4, 8, 12, and 16).
2. Lung function: including the forced expiratory volume in the first second (FEV_1_), the ratio of forced vital capacity occupied by forced expiratory volume in the first second (FEV_1_/FVC), and daytime and nighttime peak expiratory flow will be measured (at weeks 4, 8, and 16).
3. Average number of self-reported as-needed medication uses (at weeks 4, 8, 12, and 16).
4. Curative effect of TCM syndrome (at weeks 4).

Exploratory outcome: Changes in the gut microbiota composition in stools (at weeks 4). To observe the influence of XQL decoction on the composition of the gut microbiota, 16S ribosomal ribonucleic acid (16S rRNA) sequencing will be used to detect bacterial taxa present in stool samples.

### 3.4 Safety assessment

In China, XQL decoction has been used for nearly 2000 years, and the dosage used in this study is within the recommended range based on the People's Republic of China Pharmacopeia (2015 edition). Moreover, we will employ a series of measures, including subjective description and laboratory tests that especially focus on gastrointestinal intolerance, heart, liver, and kidney damage to assess the safety of XQL decoction, from the time of enrollment through the follow-up period.

### 3.5 Compliance

Once patients have been randomized, researchers at the study sites will make every reasonable effort to follow the patient for the duration of the study. At each visit, adherence to intervention will be monitored and participants will be asked to return all study containers with any unused packs of granules, including all empty containers. All examination and transportation costs will be covered and the results of physical examinations will be explained at every visit. Prior to every visit, messages will be sent through WeChat or by phone to remind patients of the upcoming data collection. In addition, ongoing support, such as free registration and treatment advice, will be provided to the participants in the follow-up phase.

### 3.6 Adverse events

Any adverse events will be recorded in CRFs irrespective of their relationship to the study intervention. In case of any serious adverse events, the intervention will be immediately stopped and a detailed description of the time, severity, relationship with the drug, and the measures taken based on standard operational procedures of the China Food and Drug Administration will be recorded. In addition, serious adverse events will be reported to the Steering Committee and Ethics Committee within 24 hours.

### 3.7 Data management and quality control

All records will be collected in CRFs, which will be completed by a trained and qualified investigator. Once a CRF is completed, the original record will not be changed if any corrections are made. The completed CRFs will be reviewed by the clinical inspector. Data entry and management will be guided by medical statistics experts. To ensure the accuracy of the data, 2 data administrators will input and proofread the data independently. After reviewing and confirming that the established database is correct, the data will be locked by the main researchers and statistical analysts. The locked data or files will not be changed thereafter and will be submitted for statistical analysis by the research group. The Sichuan TCM evidence-based Medicine Center (Chengdu, China), which does not have any competing interests, will be responsible for monitoring the data. The Department of Science Research of the hospital at Chengdu University of TCM, which is independent of the investigators, will perform data audits in the middle of the trial.

### 3.8 Statistical analysis

All data analyses will be conducted according to the intention to treat principle. Missing values will be replaced by the last observation carried forward method. Two similar participants with complete data will be double-checked to make sure that data are correct before analysis.

The data will be analyzed using the Statistical Package for the Social Sciences version 22.0 (SPSS 22.0, Chicago, IL). The analytical methods will be selected according to the distribution characteristics of the data: the measurement data will be examined using group *t* tests or nonparametric tests, the count data will be tested using a Chi-square test or Fisher exact probability method, and the grade data will be tested using nonparametric tests. Compared with the baseline values, the measurement data will be assessed using paired *t* tests or nonparametric tests, and the count data will be examined using a nonparametric test. All statistical tests will be bilateral tests and *P*-values < .05 will be considered to indicate statistical significance.

## 4 Discussion

The RA is a major public health problem that seriously affects the quality of life and even endangers the life of patients. Current available medications for chronic RA are not satisfactory and improvements in the management of RA are needed. CHM has been widely used in the treatment of asthma in China for a long time because of its low probability of adverse reactions and low price; XQL decoction is the most commonly used herbal medicine. However, to date, there is no unified understanding of the efficacy and safety of XQL decoction in the treatment of RA. Thus, a prospective and adequately powered trial is needed to conclusively determine the risks and benefits of adding XQL decoction to the treatment of patients with RA, according to syndrome differentiation.

To the best of our knowledge, this is the first clinical study to investigate the efficacy of XQL decoction plus western medicine in the treatment of RA. We are focusing on ACT, lung function, quality of life and safety, not only for the treatment phase but also during a 3-month follow-up phase. In our study, we will employ validated objective tools such as ACT scores and lung function tests. These measurements improve the reliability and generality of the results. Meanwhile, several studies have indicated a significant correlation between asthma symptoms and specific microbial species in the intestine.^[24,25]^ In this clinical trial, we will also investigate changes in the fecal microbial composition of patients after treatment with CHM and explore the possible mechanism of XQL decoction in the treatment of RA from the perspective of intestinal microecology.

There are limitations to this study. First, because the study is being performed in Sichuan, China, it is unclear whether the relative effects of the trial drugs would be similar in other ethnic groups. Second, the follow-up period is relatively short. Nevertheless, the results should contribute to the decision-making process of RA treatment and management, and should provide significant information that can be incorporated into future treatment guidelines. In the future, a multicenter randomized controlled trial with a large sample of patients with RA and the implementation of multidimensional comprehensive evaluations should be performed.

## Acknowledgments

The authors are grateful to the Sichuan Science and Technology Program for funding this study. They also thank Editage (www.editage.cn) for English language editing.

## Author contributions

**Conceptualization:** Hongjing Yang, Qingsong Huang.

**Investigation:** Jun Chen, Keni Zhao, Jianying Wu.

**Qingsong Huang orcid:** 0000-0003-1878-3579.

**Supervision:** Wei Xiao, Xiaohong Xie.

**Writing – original draft:** Wenfan Gan, Yang Yang.

**Writing – review & editing:** Chuantao Zhang, Zengtao Sun.

## Supplementary Material

Supplemental Digital Content
