# Supplementary material for: A randomized controlled trial study protocol for Xiao-Qing-Long decoction in the treatment of refractory asthma: Study protocol clinical trial (spirit compliant)
Source: Medicine (Baltimore). 2020 Jan 31;99(5):e18911. doi: 10.1097/MD.0000000000018911 (PMC7004712; doi:10.1097/MD.0000000000018911)
Supplement: Supplemental Digital Content [file medi-99-e18911-s001.docx]

**Supplementary Table** Sequences of primers used for amplification and sequencing of the *F5* gene

| Region | Primer sequence (5’→3’) | Product size  (bp) | Anealing temperature  (℃) |
| --- | --- | --- | --- |
| Exon 1 | F: CTCATTGCAGCTGGGACAG  R: GGGTGCTTTCTAAACCCTCA | 472 | 59.9 |
| Exon 2 | F: CTGTGGACTTCTGAAATACAATAAAGA  R: CACTGGGCCCCACATATTA | 384 | 59.2 |
| Exon 3 | F: TGCCTTTGATGACCCTGAAT  R: CCAGTTGCAAGAGATTTCCCTA | 291 | 60.5 |
| Exon 4 | F: TGCATCTCCAGAGAACTCATCTT  R: ATGCTCCCAAGCTTTGTCC | 399 | 60.3 |
| Exon 5 | F: GCGTCTTCTATCTGCAGTGCT  R: TTGAAGAAAACAGGACCGAAA | 263 | 59.8 |
| Exon 6 | F: TGCTTGTTTGCTGGTCACTC  R: AAAGGGCAAGGGAGAAAGAG | 497 | 59.9 |
| Exon 7 | F: TGCCTGAGCATTACTTTCACTC  R: CCCCTTGAGAGCTGTGAACT | 396 | 59.5 |
| Exon 8 | F: TCAGAAGTTTCCAGTTTTCCAAC  R: TCTCCCATGATTCTGTATTTGTGT | 394 | 59.8 |
| Exon 9 | F: CACACACACCATCCAAAAGG  R: GAAAAATGTGGCAGCCTCTC | 262 | 59.8 |
| Exon 10 | F: TAATTGGTTCCAGCGAAAGC  R: CCCCATTATTTAGCCAGGAGA | 390 | 60.3 |
| Exon 11 | F: TCCATTGGTCTATGCGTCTG  R: CAACCACAGGAATGAAAAACTG | 255 | 59.6 |
| Exon 12 | F: ATGCCCTTTTGTTGGCATAG  R: CTGGTAGCCTGGAGAGTTGC | 351 | 60.0 |
| Exon 13-1 | F: TCCCAGACTTCCAGATCTCTC  R: CTGTGACATCTGGCTGTAGAGG | 605 | 59.2 |
| Exon 13-2 | F: CCAGCCCATATTCTGAAGACC  R: TCGTGTCTTAATGAGAAACTGGC | 608 | 60.8 |
| Exon 13-3 | F: TCTACAAGTAAGACAGGATGGAGG  R: GTTCTGGAGAGAGAGTCGTGTG | 626 | 59.0 |
| Exon 13-4 | F: CAAGTCCTTCCCCACAGATATA  R: AGATCTGCAAAGAGGGGCAT | 702 | 59.6 |
| Exon 13-5 | F: CTTCTGAATCTAGTCAGTCATTGC  R: TTCAGCAGTAATGGAAAAATGAG | 450 | 57.9 |
| Exon 14 | F: CTGACCTCATGGCACTTATACC  R: CCGAAGATCTTAGCAGTGCTC | 615 | 58.9 |
| Exon 15 | F: GGCCATATCTCACAGGATGG  R: GTCATCTGAAGAGCTGCATGG | 600 | 60.8 |
| Exon 16 | F: AGTGCATGGTAAGCACTTGG  R: ACCTGCCAGATTACATCAGC | 761 | 58.3 |
| Exon 17 | F: CCTTTCCATGGCTAGGTAGG  R: TCTTAGCAGGGACCTCTTCC | 356 | 58.6 |
| Exon 18 | F: GAAAGCCTCTTGTGAAGCAGG  R: TTCAATGCAATCAGACCATGG | 388 | 61.6 |
| Exon 19 | F: ATTGAGTCAGAAACATAATCCC  R: GCATGCTGCACAACTGTAGG | 222 | 57.7 |
| Exon 20 | F: AAGGATCTGGTTTTCCACTGG  R: ACCTCAGAGGGTTGATTTTAAGG | 366 | 60.1 |
| Exon 21 | F: GCAGTGTGTGACTTGTTGAC  R: AGATTCAGATAGAAATATGCACAC | 241 | 54.8 |
| Exon 22 | F: TCTTCCTGGAACTGGAATTATCC  R: TCTTGATTCTTTGAGTGGCAGTG | 360 | 61.2 |
| Exon 23 | F: TGAGAACAGTATTTGGCACTTGG  R: CCAGATCCTCCATGTTTGTGG | 398 | 62.0 |
| Exon 24 | F: AAGCAAAGGTTTTAACATCTTCC  R: TCTTTGCCCAGATGCCAC | 258 | 59.4 |
| Exon 25 | F: CAGTCATACAGCTAATACAGACG  R: GGTCTTAAAGAGTCTCTTCCAGG | 630 | 56.1 |

F, forward; R, reverse.
